# Clinical value of the systemic immune-inflammation index in moyamoya disease

**DOI:** 10.3389/fneur.2023.1123951

**Published:** 2023-04-20

**Authors:** Erheng Liu, Chengyuan Liu, Lide Jin, Hu Zhou, Xueyi Tan, Guibo Zhang, Weihua Tao, Xiang Gao, Heng Zhao, Chao Luo, Xuehua Li, Shuaifeng Yang

**Affiliations:** ^1^Department of Neurosurgery, The Affiliated Hospital of Kunming University of Science and Technology, Kunming, China; ^2^Department of Neurosurgery, The First People's Hospital of Yunnan Province, Kunming, China; ^3^Department of AIDS/STDs Prevention and Control, Yunnan Center for Disease Control and Prevention, Kunming, Yunnan, China

**Keywords:** moyamoya disease, immune, inflammation, systemic immune-inflammation index, neutrophil/lymphocyte ratio, platelet/lymphocyte ratio

## Abstract

**Background:**

Moyamoya disease (MMD) is a rare cerebrovascular disorder with unknown etiology. The underlying pathophysiological mechanism of moyamoya disease remains to be elucidated, but recent studies have increasingly highlighted that abnormal immune response may be a potential trigger for MMD. Neutrophil-to-lymphocyte ratio (NLR), platelet-to-lymphocyte ratio (PLR), and systemic immune-inflammation index (SII) are inflammatory markers that can reflect the immune-inflammation state of the disease.

**Objective:**

The purpose of this study was to investigate SII, NLR, and PLR in patients with moyamoya disease.

**Methods:**

A total of 154 patients with moyamoya disease (MMD group) and 321 age- and sex-matched healthy subjects (control group) were included in this retrospective case–control study. Complete blood count parameters were assayed to calculate the SII, NLR, and PLR values.

**Results:**

The SII, NLR, and PLR values in the moyamoya disease group were significantly higher than those in the control group [754 ± 499 vs. 411 ± 205 (*P* < 0.001), 2.83 ± 1.98 vs. 1.81 ± 0.72 (*P* < 0.001), and 152 ± 64 vs. 120 ± 42 (*P* < 0.001), respectively]. The SII in the medium-moyamoya vessels of moyamoya disease was higher than that in the high-moyamoya vessels and low-moyamoya vessels (*P* = 0.005). Using the receiver operating characteristic (ROC) curve analysis to predict MMD, the highest area under the curve (AUC) was determined for SII (0.76 for SII, 0.69 for NLR, and 0.66 for PLR).

**Conclusion:**

Based on the results of this study, patients with moyamoya disease admitted for inpatient care due to acute or chronic stroke have significantly higher SII, NLR, and PLR when compared to blood samples drawn from completely healthy controls in a non-emergent outpatient setting. While the findings may suggest that inflammation plays a role in moyamoya disease, further studies are warranted to corroborate such an association. In the middle stage of moyamoya disease, there may be a more intense imbalance of immune inflammation. Further studies are needed to determine whether the SII index contributes to the diagnosis or serves as a potential marker of an inflammatory response in patients with moyamoya disease.

## Introduction

Moyamoya disease is a non-atherosclerotic cerebrovascular structural abnormality first described by Japanese scholars Takeuchi and Shimizu in 1957 (1). Moyamoya disease is characterized by progressive stenosis or occlusion of the internal carotid artery and the intracranial portion of its proximal branches, leading to ischemic or hemorrhagic stroke with a high disability rate and even death (2). MMD was more common in East Asian countries, such as Japan, Korea, and China, and according to a report, the annual incidence of MMD was 1.14 per 100,000 inhabitants in China from 2016 to 2018, and it increased year by year (3). The underlying pathophysiological mechanism of moyamoya disease remains to be elucidated, but recent studies have increasingly highlighted that an abnormal immune response may be a potential trigger for MMD (4–8). For example, histopathological examination of intracranial vessels in patients with moyamoya disease revealed smooth muscle hyperplasia with infiltrating macrophages and T cells in the vessel wall, and immunohistochemical studies also showed abnormal expressions of IgG and S100A4 proteins in vascular smooth muscle cells (2, 8, 9). An upregulated expression of various inflammatory factors, such as monocyte chemoattractant protein-1, interleukin-1β, and stromal cell-derived factor-1α, was found in the plasma of patients with moyamoya disease (10–12). A large cohort study of moyamoya disease also showed that autoimmune disease and infection may be potential environmental triggers (13, 14). In conclusion, increasing molecular and clinical evidence revealed that immune-inflammation-related responses may be an essential triggering factor of moyamoya disease, just below the genetic factor (15–18).

Recently, platelet-lymphocyte ratio (PLR) and neutrophil-lymphocyte ratio (NLR), as new easily available and economical biomarkers of systemic inflammation, have been confirmed in a variety of immune-inflammation diseases, including moyamoya disease (5, 19, 20). When there is no obvious infection, PLR and NLR are considered to be indicators of systemic inflammation (21–23). Studies have shown that the combination of NLR and PLR can predict mucosal diseases more accurately than NLR or PLR alone. Therefore, Hu et al. (21) proposed a comprehensive index SII based on peripheral lymphocytes, neutrophils, and platelet counts. The SII value was calculated as follows: platelet count × (neutrophil/lymphocyte). In recent years, the research field of SII has been expanding constantly. SII can not only be used as a predictor of cancer outcomes but is also associated with the severity of diseases such as acute pancreatitis and ulcerative colitis (UC) (21, 24–27). Compared with PLR and NLR, SII may better reflect the systemic immune-inflammation state as a simple, convenient, easily available, cheap, and non-invasive marker. To date, there is no report on the SII of moyamoya disease, and little is known about its clinical significance. Considering that moyamoya disease may be a disease in which both inflammation and the immune system participate, we explored the clinical value and significance of SII, NLR, and PLR in patients with moyamoya disease.

## Materials and methods

**Figure 1:**
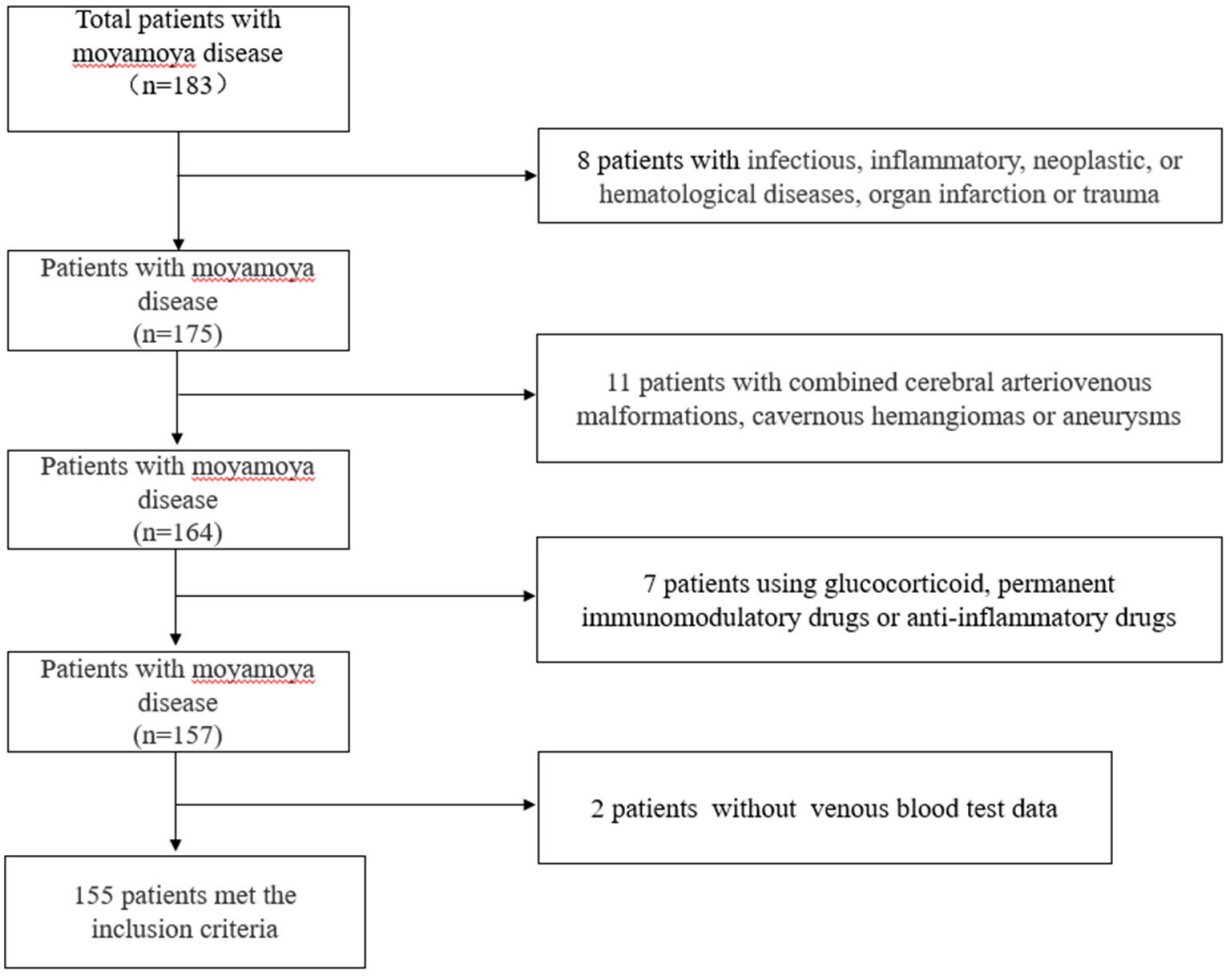
Patient inclusion flow chart.

**Figure 2:**
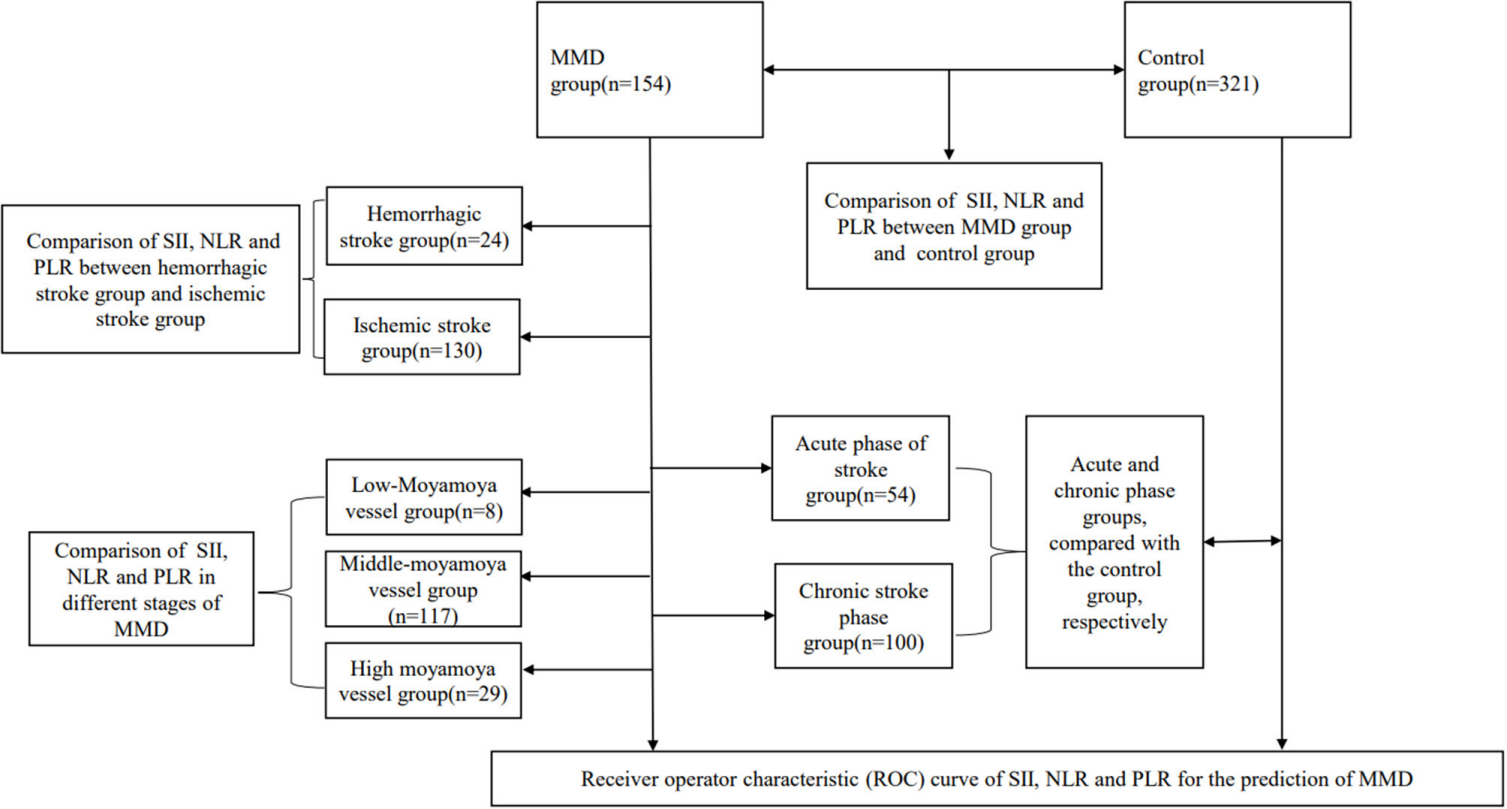
Experimental flow chart.

We retrospectively analyzed 154 inpatients diagnosed with moyamoya disease in the department of neurosurgery, at the First People's Hospital of Yunnan Province, from January 2015 to January 2021. In the case group, it was confirmed by medical history and imaging that all patients with moyamoya disease had experienced or were experiencing stroke events. The control group was selected from the population undergoing routine physical examination in our hospital. We first excluded those who had any comorbidities or routine drug use, and then, we performed age–sex matching in healthy people. Finally, 321 healthy people were randomly selected from the matched population. Blood data of participants in the control group were obtained from the physical examination center of our hospital. Our hospital is located in the southwest of China, one of the regions with a high incidence of moyamoya disease. The hospital is one of the largest stroke centers in Yunnan Province, China, and is a national Grade 3A hospital. The inclusion criteria were as follows. The cerebral angiography must show at least the following findings: (1) stenosis or occlusion of the distal portion of the intracranial internal carotid artery (ICA) or the proximal portion of the anterior cerebral artery (ACA) and/or middle cerebral artery (MCA); (2) abnormal vascular network near arterial occlusion or stenosis lesions; and (3) bilateral as found in (1) and (2). The exclusion criteria were as follows: (1) a history of infectious, inflammatory, neoplastic, or hematological diseases, organ infarction, or trauma; (2) combined cerebral arteriovenous malformations, cavernous hemangiomas, or aneurysms; (3) atherosclerosis; (4) patients treated with glucocorticoid, permanent immunomodulatory drugs, or anti-inflammatory drugs; (5) autoimmune diseases, and (6) a lack of venous blood test data. The patient inclusion flow chart is shown in Figure 1. Angiographic examinations were performed by two neuroradiologists. The stage of Suzuki was determined by digital subtraction angiography (DSA). According to the Suzuki stage, patients with moyamoya disease were divided into three subgroups: high-moyamoya vessels, medium-moyamoya vessels, and low-moyamoya vessels. High-moyamoya vessels are defined as Grades V and VI of Suzuki, medium-moyamoya vessels are defined as Grades III and IV of Suzuki, and low-moyamoya vessels are defined as Grades I and II of Suzuki. According to the medical history and imaging data, patients were divided into an acute stage group (stroke time < 1 month) and a chronic stage group (stroke time ≥ 1 month). If on admission, imaging results show that the patients are in the acute or subacute stage of stroke, regardless of their stroke history, they are still grouped in the acute stage of stroke. All samples were collected at 8 a.m. on the 2nd day of admission, and blood samples were collected from fasting venous blood. The flow chart of the research scheme is shown in Figure 2. We obtained informed consent from the participants in the control group. This study was approved by the Clinical Research Ethics Committee of the First People's Hospital of Yunnan Province (2018LH118), which determined that informed consent was exempted for the moyamoya patient group and that its protocol conformed to the principles of the Declaration of Helsinki.

### Demographic and laboratory data

Demographic and clinical information on admission was collected retrospectively for all patients, and we included the following data: (1) baseline data: sex, age, risk factors for cerebrovascular diseases (such as hypertension, diabetes, and hyperlipidemia), and personal history (such as smoking and drinking history); (2) clinical laboratory parameters at the time of hospitalization: neutrophils, lymphocytes, monocytes, white blood cells (WBC), platelet count, and calculation of NLR, PLR, and SII; and (3) stroke type, Suzuki stage, and stroke period. The NLR was calculated by dividing the neutrophil count by the lymphocyte count, the PLR was calculated by dividing the platelet count by the lymphocyte count, and SII was calculated as platelet count × (neutrophil/lymphocyte).

### Statistical analysis

The measurement data follow a normal distribution and are expressed by the mean standard deviation (mean ± SD). The means of two continuous normally distributed variables were compared by independent samples using the Student's *t*-test or ANOVA. The area under the curve (AUC) was calculated according to the receiver operating characteristic curve (ROC) of probability mapping, and the diagnostic efficiency of each parameter was evaluated. Statistical analysis was performed using SPSS 20.0 software (IBM SPSS Inc, Chicago, IL, United States). A *p*-value of < 0.05 was considered statistically significant

## Results

The study included 154 patients with moyamoya disease and 321 age- and sex-matched healthy subjects as the control group. The mean ages of patients in the moyamoya disease group and the control group were 47 ± 12 years old and 48 ± 11 years old, respectively (*P* = 0.323). Gender was also similar between the two groups (*P* = 0.945). SII, NLR, and PLR levels were significantly higher in the moyamoya disease group compared with the control group [754 ± 499 vs. 411 ± 205 (*P* < 0.001), 2.83 ± 1.98 vs. 1.81 ± 0.72 (*P* < 0.001), and 152 ± 64 vs. 120 ± 42 (*P* < 0.001), respectively; Table 1]. In order to reduce the interference in the acute phase of stroke, we further divided the moyamoya disease group into the acute phase subgroup and the chronic phase subgroup according to the stroke period. There were 54 cases (35.1%) in the acute phase subgroup and 100 cases (65.9%) in the chronic phase subgroup, which were then compared with the control group, respectively. The results showed that in the acute phase subgroup, the differences of SII, NLR, and PLR were significant, and the differences in the chronic phase subgroup were also significant [acute phase subgroup: SII (1,082 vs. 411, *P* < 0.001), NLR (4.30 vs. 1.81, *P* < 0.001), PLR (166 vs. 120, *P* < 0.001); chronic phase subgroup: SII (577 vs. 411, *P* < 0.001), NLR (2.01 vs. 1.81, *P* < 0.001), PLR (145 vs. 120, *P* < 0.001); Table 2]. Further analysis was performed within the moyamoya group. The moyamoya disease patients were divided into a hemorrhagic subgroup and an ischemic subgroup according to the cranial MRI results and medical history, of which 24 cases (15.6%) were in the hemorrhagic subgroup, and 130 cases (84.4%) were in the ischemic subgroup. SII, NLR, and PLR levels were significantly higher in the hemorrhagic subgroup compared with the ischemic subgroup (SII with *P* < 0.001, NLR with *P* < 0.001, and PLR with *P* = 0.02; Table 3). According to the Suzuki stage, the moyamoya disease group was divided into low-, medium-, and high-moyamoya vessel subgroups: eight cases (5.1%) were in the low-moyamoya vessel subgroup, 117 cases (75.9%) were in the middle-moyamoya vessel subgroup, and 29 cases (19%) were in the high-moyamoya vessel subgroup. The results showed that only SII had significant differences in the three subgroups [SII (482 vs. 824 vs. 535, *P* = 0.005), NLR (2.40 vs. 2.98 vs. 2.36, *P* = 0.265), and PLR (153 vs. 158 vs. 130, *P* = 0.108); Table 4]. To explore whether the SII index was different in different stages of moyamoya disease, we further analyzed the results and found that the SII in the middle-moyamoya vessel subgroup was significantly higher than that in the low- and high-moyamoya vessel subgroups (*P* = 0.005).

**Table 1:** Comparison of demographic features and clinical characteristics between the study and control groups.

**Variables**	**Moyamoya disease group (*n* = 154)**	**Control group (*n* = 321)**	***P*-value**
Age (years), mean ± SD	47 ± 12	48 ± 11	0.323
Gender (male), *n* (%)	70 (45.5)	147 (45.8)	0.945
Neutrophil count ( × 10^9^/L)	4.90 ± 2.31	3.42 ± 1.17	**< 0.001**
Lymphocyte count ( × 10^9^/L)	1.99 ± 0.71	2.02 ± 0.60	0.660
Platelet count ( × 10^9^/L)	273.32 ± 71.18	226.75 ± 57.40	**< 0.001**
SII	754 ± 499	411 ± 205	**< 0.001**
NLR	2.83 ± 1.98	1.81 ± 0.72	**< 0.001**
PLR	152 ± 64	120 ± 42	**< 0.001**

Values are mean ± SD. Boldface-type indicates statistical significance with a P-value of < 0.05.

SII, systemic immune-inflammation index; NLR, neutrophil-to-lymphocyte ratio; PLR, platelet-to-lymphocyte ratio.

Student's t-test or chi-square test.

**Table 2:** Moyamoya disease group was divided into acute and chronic phase groups and compared with the control group, respectively.

**Biomarker**	**Stroke period**	**Number of cases**	**Mean value**	***t***	***P*-value**
SII	Acute phase of stroke	54	1,082	15.457	**< 0.001**
	Chronic phase of stroke	100	577	6.051	**< 0.001**
	Control group	321	411		
NLR	Acute phase of stroke	54	4.30	14.591	**< 0.001**
	Chronic phase of stroke	100	2.04	2.644	0.009
	Control group	321	1.81		
PLR	Acute phase of stroke	54	166	6.709	**< 0.001**
	Chronic phase of stroke	100	145	4.432	**< 0.001**
	Control group	321	120		

Boldface type indicates statistical significance with a P-value of < 0.05.

SII, systemic immune-inflammation index; NLR, neutrophil-to-lymphocyte ratio; PLR, platelet-to-lymphocyte ratio.

Student's t-test.

**Table 3:** Moyamoya disease group was divided into the hemorrhagic stroke group and the ischemic stroke group.

**Biomarker**	**Stroke type**	**Number of cases**	**Mean value**	***t***	***P*-value**
SII	Ischemic stroke	130	682	−4.391	**0.000**
	Hemorrhagic stroke	24	1,143		
NLR	Ischemic stroke	130	2.60	−3.563	**0.000**
	Hemorrhagic stroke	24	4.11		
PLR	Ischemic stroke	130	147	−2.348	**0.020**
	Hemorrhagic stroke	24	180		

Boldface type indicates statistical significance with a P-value of < 0.05.

SII, systemic immune-inflammation index; NLR, neutrophil-to-lymphocyte ratio; PLR, platelet-to-lymphocyte ratio.

Student's t-test.

**Table 4:** Comparison of biomarkers in different stages of moyamoya disease.

**Biomarker**	**Stages of moyamoya disease**	**Number of cases**	**Mean value**	**Unadjusted 95% CI**	***F***	***P*-value**
SII	Low-moyamoya vessel	8	482	(192–772)	5.534	**0.005**
	Middle-moyamoya vessel	117	827	(728–925)		
	High moyamoya vessel	29	535	(451–618)		
NLR	Low-moyamoya vessel	8	2.40	(0.54–4.25)	1.341	0.265
	Middle-moyamoya vessel	117	2.98	(2.59–3.36)		
	High moyamoya vessel	29	2.36	(1.92–2.81)		
PLR	Low-moyamoya vessel	8	153	(117–189)	2.257	0.108
	Middle-moyamoya vessel	117	158	(145–170)		
	High moyamoya vessel	29	130	(115–145)		

Boldface type indicates statistical significance with a P-value of < 0.05.

CI, confidence interval; SII, systemic immune-inflammation index; NLR, neutrophil-to-lymphocyte ratio; PLR, platelet-to-lymphocyte ratio.

Analysis of variance.

**Figure 3:**
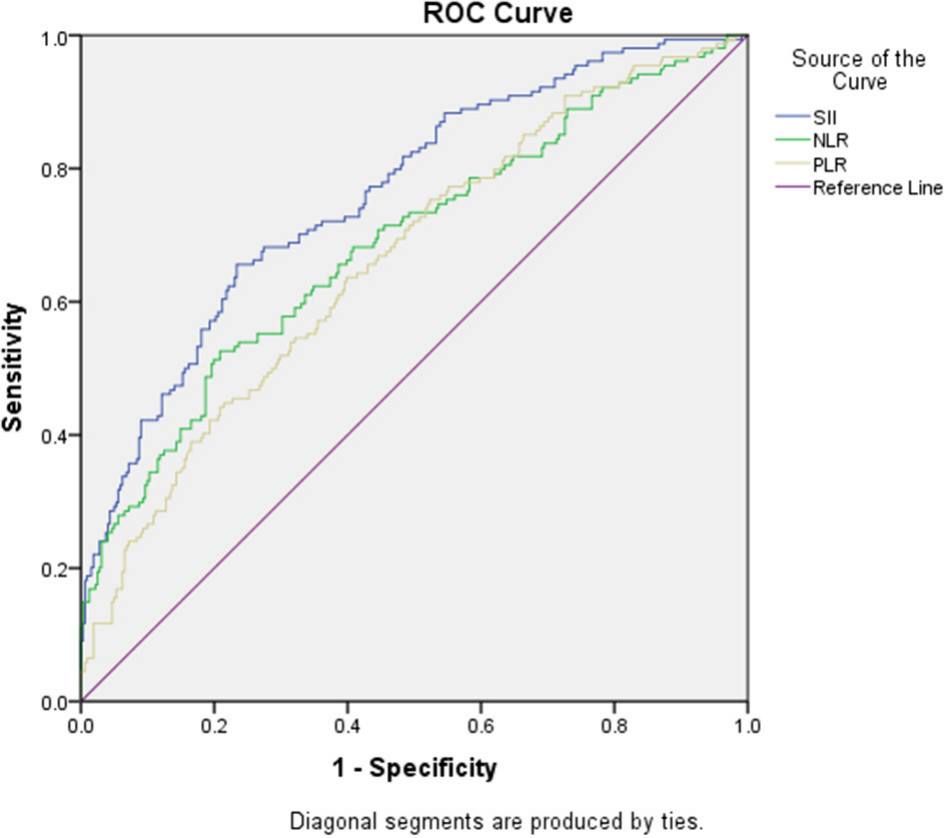
Receiver operating characteristic curve.

According to the ROC curve analysis, the predictive value of the SII, NLR, and PLR was evaluated by comparing the AUC area. The AUC of the SII, NLR, and PLR for MMD was 0.76, 0.69, and 0.66, respectively (Figure 3 and Table 5).

**Table 5:** Receiver operator characteristic (ROC) curve of the systemic immune-inflammation index (SII), neutrophil/lymphocyte ratio (NLR), and platelet-to-lymphocyte ratio (PLR) for the prediction of moyamoya disease.

**Biomarker**	**AUR**	**Standard error**	***P*-value**	**95%CI**
SII	0.76	0.02	< 0.001	(0.71–0.81)
NLR	0.69	0.03	< 0.001	(0.63–0.74)
PLR	0.66	0.03	< 0.001	(0.61–0.71)

CI, confidence interval.

## Discussion

Based on the results of this study, patients with moyamoya disease admitted for inpatient care due to acute or chronic stroke have a significantly higher SII, NLR, and PLR when compared to blood samples drawn from completely healthy controls in a non-emergent outpatient setting. Furthermore, we found that SII levels in patients with intermediate-moyamoya vessel subgroups were higher than those in low- and high-moyamoya vessel subgroups. Our results suggest that there may be different active phases of immune inflammatory responses at different stages of moyamoya disease. This may further explain why patients with mid-stage MMD are more likely to suffer from strokes. Compared to other inflammatory markers, the SII is a new and beneficial tool, and it may be a reliable, universal, and cheap inflammatory index in moyamoya patients.

Moyamoya disease has long been a disease of unknown etiology. At present, more and more research indicates that moyamoya disease is a multi-factor disease (4, 5). Studies on MMD in many aspects, such as immune factors, immune cells, immune genes, and immune diseases, have hinted that it is related to the occurrence and development of MMD. It is now increasingly recognized that moyamoya disease is a synergistic effect of genetic and environmental risk factors such as infection, inflammation, and autoimmunity (2, 17, 28). As early as 2004, when analyzing the cerebrospinal fluid of patients with moyamoya disease, Kim et al. (29). found that autoantibodies commonly existed in the cerebrospinal fluid of patients with MMD, including immunoglobulin G, the CD163 subgroup heavy protein, and CD40 molecular recombinant protein. A CD40-mediated inflammatory response may be a factor for vascular wall-induced stenosis (30). CD163 is a CD163-positive M2-polarized macrophage activation marker, which has been confirmed to be related to various immune diseases (8). IgG autoantibodies in the serum of patients with moyamoya disease were also significantly increased (30). In immune cells, the occurrence and development of moyamoya disease may be related to the imbalance of the ratio of initial B cells, initial CD4 cells, resting natural killer cells, and regulatory T cells (7, 30). The RNF213 gene was identified as the major susceptibility gene for MMD. The RNF213 gene is associated with immune function and plays an important role in antigen uptake, processing, and presentation (31–33). The differential expression of abnormal immune-related genes also suggested that MMD might show immune-related gene disorder in peripheral blood (34–36). Although little is known about the biological function of RNF213 or its related pathogenesis in moyamoya disease, the identification of RNF213 as an immunosensor reveals a clear molecular link between MMD and infection (31, 36, 37). In recent years, increasing reports have been made about the coexistence of moyamoya disease and immune diseases, such as Graves' disease and systemic lupus erythematosus (38–41). In the United States and China, patients with moyamoya disease combined with autoimmune diseases have been reported to have a higher prevalence than the general population, but the exact reason is unclear (42, 43). Patients with moyamoya disease were considered to have a similar history of infection, such as varicella-zoster virus, cytomegalovirus, and EBV infection (44–47). COVID-19-mediated immune disorder and cytokine storm can promote and aggravate moyamoya vascular disease and affect its course (48). It has been proposed that chronic inflammation in the autoimmune process may be the trigger for moyamoya disease, but systemic acute inflammatory diseases may also be involved in the development of moyamoya disease (49). In summary, the existing evidence strongly suggests that immune inflammation may be a very important factor in the development of moyamoya disease, playing a very important role in the pathogenesis of moyamoya disease.

SII is a new, inexpensive biomarker that can be easily calculated using platelet, neutrophil, and lymphocyte counts, showing a balance between inflammation and immune response (21, 24, 25). At present, SII shows very high reliability in reacting to the body's immune-inflammation state in a plurality of diseases, such as predicting tumor recurrence and activity of ankylosing spondylitis, as well as bell palsy and irritable bowel syndrome (50–53). Interestingly, the systemic immune-inflammation index was also associated with increased carotid intima-media thickness in hypertensive patients (54). Although moyamoya disease is primarily a proliferative disease of the arterial lining, usually not infiltrating with inflammatory cells, studies have reported the presence of T cells in moyamoya vascular specimens from necropsy in patients with moyamoya disease (55, 56). The role of inflammation in the thickening of intimal fibroblasts and the pathogenesis of the disease is still under investigation. At present, there are few studies on the normal range of SII, PLR, and NLR, but studies have shown that PLR and NLR are race-specific (57–59). To avoid race-specific effects, our study used our hospital's healthy subjects as the normal healthy control group.

In the results of this study, the SII, PLR, and NLR indexes of patients with moyamoya disease were higher than those of the healthy control group, with significant statistical significance. In order to reduce the stress response of the body by itself in the acute phase, the moyamoya disease group was further divided into the acute subgroup and chronic subgroup. The SII in the chronic subgroup was still higher than that in the control group. Based on the results of this study, patients with moyamoya disease may have a low-grade immune inflammatory state, and there may be an imbalance between neutrophils, lymphocytes, and platelets. Our results are consistent with the idea that inflammation and immune response may be important factors in moyamoya disease. When we classified moyamoya disease according to the type of stroke, we found that the SII, NLR, and PLR of hemorrhagic moyamoya disease were higher than those of ischemic moyamoya disease. We cannot help but consider that it is possible that the cause of hemorrhagic stroke in patients with moyamoya disease is a more serious imbalance of immune inflammation than ischemic moyamoya disease. According to the ROC curve, SII may be advantageous over other inflammatory markers and may be a reliable, universal, and inexpensive indicator of inflammation in patients with moyamoya disease. SII may be a biomarker for predicting what type of stroke may occur in patients with moyamoya disease, which may require a large number of prospective studies in the future to prove. Interestingly, our results show that SII was higher in the middle-moyamoya vessels subgroup than in the low-moyamoya vessels subgroup and the high-moyamoya vessels subgroup. This seems to suggest that the middle stage of moyamoya disease may be a more active stage. This finding appears to imply that patients with stages III and IV moyamoya disease in the Suzuki stage are more likely to suffer a stroke. This may provide a reference for the timing of surgical intervention. Interestingly, in many studies, most stroke patients with moyamoya disease are in stages 3–4 of the Suzuki stage (60, 61). From the perspective of immune inflammation, we propose that the immune-inflammation state of the middle-moyamoya blood vessels group may become more active, while the low-moyamoya blood vessels and high-moyamoya vessels are relatively stable, which needs further investigation. This study may add new information for understanding the pathophysiological roles of immunity and inflammation in moyamoya disease. Immunoinflammatory therapy may be an important step in the treatment of moyamoya disease.

The interactions between inflammation, the immune system, and the nervous system are still unclear. Clinical analysis of immunological biomarkers of inflammation in the peripheral blood of patients with moyamoya disease may involve many aspects, including protection, injury, and nerve regeneration. These factors will make research and treatment strategies for moyamoya disease particularly complex. To sort out the complicated relationship between immune inflammation and to conduct further experimental and clinical studies related to moyamoya disease, immune inflammation will help reveal the complex role of the inflammatory immune system in the pathophysiology of MMD. In the future, it may be possible to carry out therapeutic and targeted interventions on the immune inflammatory process of moyamoya disease.

## Limitations

This study has some limitations. First, there was a relatively small sample size per group and a retrospective case–control design lacking baseline levels and dynamic changes in long-term inflammatory markers. Second, this study focused on the static values of SII at the baseline, which may not reflect the comprehensive and dynamic changes in the patient's condition. Third, considering the rarity of moyamoya disease, this study lacks prospective randomized controlled studies and does not consider other parameters (such as erythrocyte sedimentation rate, cytokine, and C-reactive protein), and the number of inflammatory factors studied is small. Fourth, although we define the chronic phase as one month after stroke, the stress response of the body may still exist, and the findings of this study may still overestimate the causal relationship and clinical relevance. Although we considered statistical bias, the findings of this study may still overestimate causality and clinical relevance. Fifth, the actual number of immune cells in the brain parenchyma, the heterogeneity of neutrophils and lymphocytes, the mechanism of immune cell release, and interactions with platelets and other components of the coagulation system were not considered. Sixth, the comparison of blood samples from hospitalized patients with MMD with acute or chronic stroke and from completely healthy patients without medical treatment in the outpatient setting makes it difficult to assess whether the differences in SII, NLR, and PLR observed between the two groups are attributable to MMD itself and therefore may contribute to outcome bias. The next step is to combine the baseline level and dynamic changes of inflammatory indicators. In the future, it may be meaningful to combine the baseline level and dynamic changes of inflammatory indicators and then use a larger sample size to assess the occurrence, development, and long-term prognostic value of the immune inflammatory system in patients with moyamoya disease.

## Conclusion

Based on the results of this study, patients with moyamoya disease admitted for inpatient care due to acute or chronic stroke have a significantly higher SII, NLR, and PLR when compared to blood samples drawn from completely healthy controls in a non-emergent outpatient setting. While the findings may suggest that inflammation plays a role in moyamoya disease, further studies are warranted to corroborate such an association. In the middle stage of moyamoya disease, there may be a more intense imbalance of immune inflammation. Further studies are needed to determine whether the SII index contributes to the diagnosis or serves as a potential marker of an inflammatory response in patients with moyamoya disease.

## Data availability statement

The original contributions presented in the study are included in the article/supplementary material, further inquiries can be directed to the corresponding authors.

## Ethics statement

This study was approved by the Clinical Research Ethics Committee of the First People's Hospital of Yunnan Province (2018LH118), which determined that informed consent was exempt for moyamoya patient group and that its protocol conformed to the principles of the Declaration of Helsinki.

## Author contributions

EL, LJ, and HuZ designed the research. XT, GZ, CLi, and WT collected and analyzed the data. XG, HeZ, and CLu drafted the manuscript. SY and XL revised the manuscript. All authors contributed to the article and approved the submitted version.
